# Supplementary material for: Communicating Mass Spectrometry Quality Information in mzQC with Python, R, and Java
Source: J Am Soc Mass Spectrom. 2024 Jun 26;35(8):1875–82. doi: 10.1021/jasms.4c00174 (PMC11311537; doi:10.1021/jasms.4c00174)
Supplement: Supplementary file 1 — js4c00174_si_001.pdf [file js4c00174_si_001.pdf]

Supporting information for "Communicating mass spectrometry quality information in mzQC with Python, R, and Java"

Chris Bielow[1,*,‡], Nils Hoffmann[2,‡], David Jimenez-Morales[3], Tim Van Den Bossche[4,5], Juan Antonio Vizcaíno[6], David L. Tabb[7], Wout Bittremieux[8], Mathias Walzer[6,*]

‡ equal contribution
* corresponding authors: chris.bielow@bsc.fu-berlin.de, walzer@ebi.ac.uk

1. Bioinformatics Solution Center, Institut für Mathematik und Informatik, Freie Universität Berlin, Takustr. 9, 14195 Berlin, Germany
2. Institute for Bio- and Geosciences (IBG-5), Forschungszentrum Jülich GmbH, 52428 Jülich, Germany
3. Department of Medicine, Stanford University School of Medicine, Stanford, CA, USA
4. Department of Biomolecular Medicine, Faculty of Medicine and Health Sciences, Ghent University, Technologiepark-Zwijnaarde 75, 9052 Ghent, Belgium
5. VIB-UGent Center for Medical Biotechnology, VIB, Technologiepark-Zwijnaarde 75, 9052 Ghent, Belgium
6. European Molecular Biology Laboratory, EMBL-European Bioinformatics Institute (EMBL-EBI), Hinxton, Cambridge, CB10 1SD, United Kingdom
7. European Research Institute for the Biology of Ageing, University Medical Center Groningen, 9713 AV Groningen, the Netherlands
8. Department of Computer Science, University of Antwerp, 2020 Antwerpen, Belgium

Supplementary Table 1: QC metrics considered during the analysis and their accession numbers in the PSI-MS controlled vocabulary. The last column refers to the programming language used to compute the metrics.

Accession	Metric	Programming Language
MS:1000505	base peak intensity	Java
MS:1002404	count of identified proteins	Python
MS:1003044	number of missed cleavages	Python
MS:1003250	count of identified peptidoforms	Python
MS:1003251	count of identified spectra	Python
MS:4000060	number of MS2 spectra	Python
MS:4000069	m/z acquisition range	Java
MS:4000070	retention time acquisition range	Java
MS:4000071	number of chromatograms	Java
MS:4000104	total ion currents	Java
MS:4000132	MS1 ion collection time mean	R
MS:4000133	MS1 ion collection time sigma	R
MS:4000137	MS2 ion collection time mean	R
MS:4000138	MS2 ion collection time sigma	R
MS:4000178	precursor ppm deviation mean	Python
MS:4000179	precursor ppm deviation sigma	Python
MS:4000180	table of missed cleavage counts	Python
MS:4000181	identified MS2 quarter RT fraction	Python
